# Enhancement of Hydrate Stability through Substitutional Defects

**DOI:** 10.1021/acs.cgd.3c00457

**Published:** 2023-06-28

**Authors:** Megan E. Fleming, Jennifer A. Swift

**Affiliations:** Department of Chemistry, Georgetown University, 37th and O Streets NW, Washington, D.C. 20057-1227, United States

## Abstract

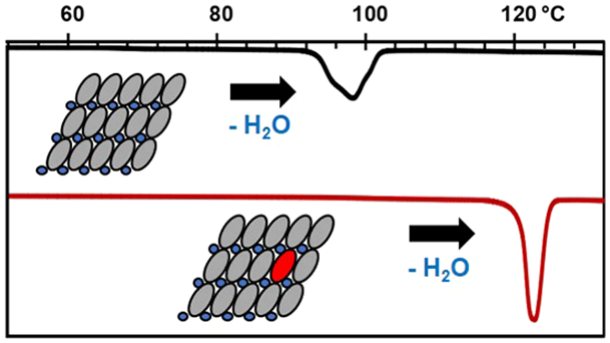

Cytosine monohydrate (CM) and anhydrate crystal forms reversibly interconvert under high temperatures or high humidity conditions. Here, we demonstrate through defect engineering the ability to expand the thermal stability range of CM through the targeted creation of quantifiable defects in low-level concentrations. Twelve different molecular dyes with a variety of core structures and charges were screened as potential dopants in CM. CM-dye phases prepared with Congo red (CR), Evans blue (EB), and Azocarmine G (AG) exhibited the highest inclusion levels (up to 1.1 wt %). In these doped isomorphous materials, each dye is presumed to substitute for 4–7 cytosine molecules within the low-rugosity (102) planes of the CM matrixes, thereby creating a quantifiable substitutional defect and an impediment to the cooperative molecular motions which enable the transformation to the anhydrate. Dehydration of materials with these engineered defects requires significantly higher temperatures and proceeds with slower kinetics compared to pure CM. The CM-dye phases also exhibit a reduction in the thermal expansion along key crystallographic axes and yield dehydration products with altered particle morphologies.

## Abstract 2

The thermal stability of cytosine monohydrate is significantly enhanced by the inclusion of select dopants in ≤1 wt %. Each included dopant molecule substitutes for 4−7 cytosine molecules within a low-rugosity (102) plane, serving as an impediment to the cooperative molecular motions which facilitate the transformation to the anhydrate.

## Introduction

Small molecule active pharmaceutical ingredients (APIs) can typically crystallize in multiple forms. This can include forms that have the same chemical composition but different lattices (polymorphs), and forms where the molecule and another component(s) cocrystallize in a periodic lattice (e.g., hydrates, solvates, and cocrystals).^1−5^ A significant fraction of APIs can crystallize as hydrates.^6,7^ Form conversions can be especially challenging with hydrates since their stability relative to water-free forms is determined by both their internal structure as well as external factors such as temperature and relative humidity. Since hydrate–anhydrate transformations can alter the properties of manufactured products,^8−10^ avoiding conditions where such phase changes are likely to occur is a significant issue for drug formulation and storage. Here we take a different approach and demonstrate the potential to rationally stabilize hydrates not through control of external factors, but rather, through more subtle internal structure modifications created by defect engineering in a well-studied model system.

The nucleobase cytosine is integral to the structure and function of DNA and RNA and it is also a substructure in a number of pharmaceuticals.^11−13^ Crystallographic studies of cytosine monohydrate (CM) and anhydrate date back to the 1960s.^14−21^ In ref (21), we reported detailed mechanistic investigations of the solid-state dehydration of CM to its dehydrated anhydrous form (Cd). Using time-resolved synchrotron powder X-ray diffraction (PXRD) to track structural changes throughout the reaction, and thermogravimetry to follow water loss, CM was found to convert directly to Cd with no other crystalline intermediates. CM and Cd share the same one-dimensional hydrogen-bonded ribbon motif though the π-stacking between ribbons is different. This led us to propose a molecular-level dehydration model wherein simultaneous water loss and ribbon-rotation provided a low-energy “switch-like” mechanism to convert between one form and the other. Recent nanomechanical studies on CM single crystals provided further support for the proposed ribbon-rotation mechanism.^22^

Building off our detailed understanding of the molecular-level dehydration mechanism, here we demonstrate the ability to rationally tune the process by engineering defects in CM that restrict the ribbon-rotation mechanism. Quantifiable defects are created through the inclusion of dye molecules in the CM structure in low-concentrations (typically ∼1 wt % or less). In the doped CM-dye materials prepared, each included dye molecule is presumed to replace 4–7 cytosine molecules within a low-rugosity (102) plane. Since dehydration induces a buckling of this plane, dopant inclusion can impose an additional barrier to the formation of Cd. Using a combination of spectroscopic, thermal, and time-resolved synchrotron PXRD methods, we assess how the substitutional defects introduced alter the thermal stability of CM, and the kinetics of its process-induced dehydration.

## Experimental Section

### Materials

**Figure 1:**
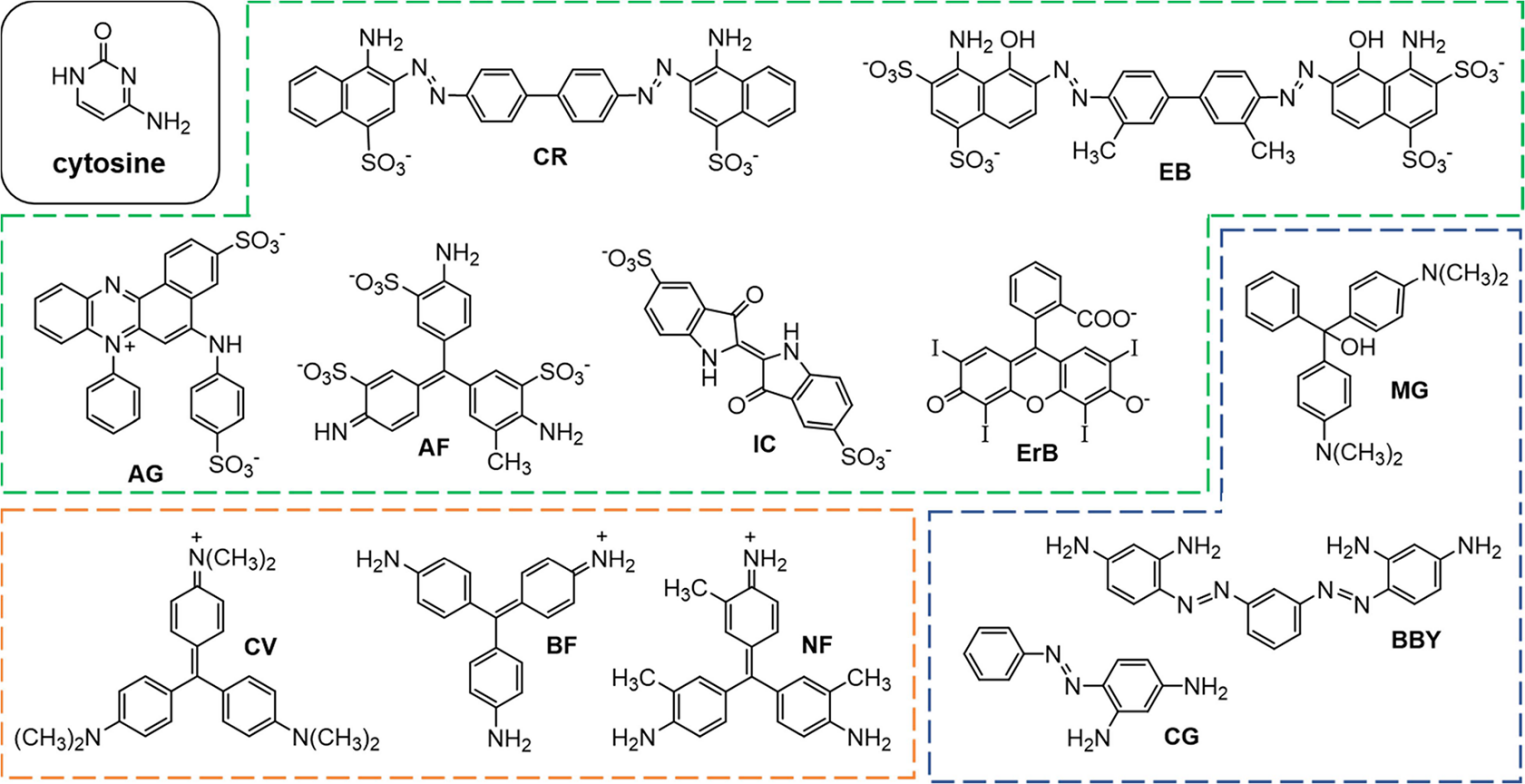
Molecular structure of cytosine and the organic dye dopants used in crystallization studies. Anionic dyes (green box): Congo Red (CR), Evans Blue (EB), Azocarmine G (AG), Acid Fuchsin (AF), Indigo Carmine (IC), and Erythrosin B (ErB). Neutral dyes (blue box): Malachite Green Carbinol hydrochloride (MG), Chrysoidine G (CG), and Bismarck Brown Y (BBY). Cationic dyes (orange box): Crystal Violet (CV), Basic Fuchsin (BF), and New Fuchsin (NF).

Cytosine was obtained from Aldrich (≥99%) and used as received. Ultrapure 18.2 MΩ deionized water from an Elga Purelab Flex purification system was used in all crystal growth solutions. All dyes shown in Figure 1 were purchased from Aldrich in the highest available purity and used as received. Acid Fuchsin (AF) [3244-88-0] 70%, Azocarmine G (AG) [25641-18-3], Basic Fuchsin (BF) [569-61-9] 96%, Bismarck Brown Y (BBY) [10114-58-6] 53%, Chrysoidine G (CG) [532-82-1] 90%, Congo Red (CR) [573-58-0] 97%, Crystal Violet (CV) [548-62-9] ≥90%, Erythrosin B (ErB) [16423-68-0] 80%, Evans Blue (EB) [314-13-6] 75%, Indigo Carmine (IC) [860-22-0] 94%, Malachite Green Carbinol hydrochloride (MG) [123333-61-9] 85% and New Fuchsin (NF) [3248-91-7] 80%.

### CM and CM-Dye Crystal Growth

CM crystals were prepared by slow evaporation of saturated aqueous cytosine solutions (4 mg/mL = 36.0 mM). The supersaturated solutions were added to Pyrex petri culture dishes (100 × 10 mm) and maintained at 25 ± 1 °C. CM crystals typically grew as rectangular plates with large (100) faces and smaller (010) and (001) side faces after 24–48 h.

Stock dye solutions (2.5 mM) were prepared. Dye-doped CM crystals were prepared similar to CM crystals, from saturated aqueous solutions with dye concentrations ranging from 0.001 to 2.5 mM (1 to 2500 μM). We refer to the dye-doped materials as CM-dye_*x*_ where *x* = the solution dye concentration in μM. The concentration range varied for each dye, but in each case, CM-dye crystals typically appeared after 24–48 h. CM-dye crystals grew in a range of morphologies.

### Microscopy

Optical micrographs were collected on an Olympus BX-50 polarizing microscope fitted with a Lumenera Xfinity 2.0 camera attachment and Xfinity Analyze software (Lumenera, Ontario). Hot stage microscopy was accomplished with an HCS302 optical hot-stage (INSTEC, Inc., Boulder, CO). Crystals were placed on 1 mm thick glass microscope slide and micrographs were collected as samples were heated from 25 to 150 °C at 5 °C/min.

Scanning electron microscopy (SEM) images were obtained on a Zeiss SUPRA55-VP microscope. CM, CM-dye, and dehydrated samples were mounted on a 3.1 mm carbon-tape layered aluminum mount (Amray Instruments). All images were taken with an in-lens detector with an acceleration voltage of 1 kV.

### Dye Quantification and Absorption in CM-Dye

The concentration of included dye in CM-dye crystals was determined using solution UV–vis spectroscopy on dissolved crystals. Calibration curves with *R*^2^ values of ∼0.99 were established by preparing standard dye solutions in a range of concentrations, typically 0.0001–0.2 mM depending on the dye solubility. CM-dye samples were hand-ground, weighed, dissolved in DI water, and their UV–vis absorbance measured on an Agilent 8453 UV–vis spectrometer with a wavelength range of 200–800 nm. Average dye concentrations in CM-dye were determined based on the calibration curve.

Solid state UV–vis data were collected on hand-ground CM-dye powders using an Agilent Cary 5000 UV–vis–NIR Spectrometer.

### Thermal Analysis

Differential scanning calorimetry (DSC) data were obtained on a TA Instruments DSC Q25. DSC experiments were performed on 3.0–5.0 mg samples in hermetically sealed aluminum pans (TA Instruments). Samples of CM and CM-dye were heated at 5.0 °C/min over the temperature range 25–130 °C. Values cited refer to triplicate measurements.

Thermogravimetric (TGA) data were obtained on a TA Instruments SDT Q600 or Q50 (New Castle, DE) using a nitrogen flow rate of 50 mL/min. All experiments used ∼3.0 mg of CM or CM-dye in open 90 μL aluminum pans (TA Instruments). Isothermal experiments were performed at 50, 55, 60, and 65 °C. The fraction dehydrated, α, at any given time was determined from the wt % loss at each data point relative to the total wt % loss (CM calc. = 13.9 wt %). The α values obtained were used in model-based and model-free kinetic analyses.

### Powder X-ray Diffraction (PXRD)

PXRD data were collected on a Rigaku Ultima IV powder diffractometer equipped with a D/teX Ultra silicon strip detector using CuKα radiation (40 kV and 44 mA current). Ground samples were prepared on zero-background silicon sample holders, with data collection from 2θ = 5–40° (2 °/min scan speed and 0.02° step size). PXRD data were analyzed using JADE and Panalytical X’Pert HighScore Plus software.^23^

PXRD data were also collected at room temperature using a DUO Apex X-ray diffractometer using Cu Kα radiation (50 kV and 30 mA current). Hand-ground powdered samples were prepared in Kapton capillaries (0.032″ ID × 0.034″ OD, Cole-Parmer) with data collection over 2θ = 5–40°. The data were integrated using APEX-2 software.

### Time-Resolved Synchrotron PXRD

Synchrotron PXRD data were collected at beamline 17-BM-B at the Advanced Photon Source (APS). Experiments had an X-ray beam λ = 0.4539 Å and a beam size of 300 μm. The beamline uses a Si (311) monochromator, a Perkin Elmer a-Si Flat Panel PE1621 area detector, and an Oxford Cryosystems Cryostream 700+. Samples were ground in the mother liquor, loaded wet into Kapton capillaries (1.1 mm OD, Cole-Palmer), and stoppered with glass wool. Capillaries were then placed in a flow cell designed for in-situ experiments.^24^

In dehydration experiments at 0% RH, dry He (5 mL/min) flowed continuously while the sample was heated at 10 K/min. Samples were rocked at 15° throughout the data collection. Exposure time was 1–2.0 s/image summed over 10 images, enabling the collection of a high Q-range PXRD pattern every 13–20 s. GSAS-II software^25^ was used for data processing and 2D image integration. Pawley refinements were done in TOPAS-V6.^26^ Size and strain parameters were refined for each data set and the parameters that afforded the lowest *R*_wp_ were used in thermal expansion plots.

## Results and Discussion

**Figure 2:**
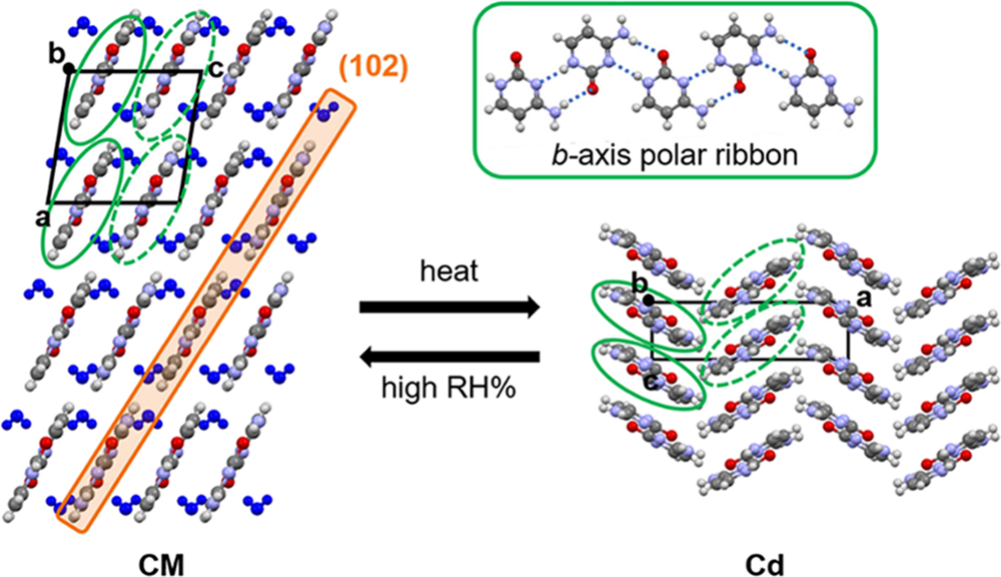
Packing diagram of cytosine monohydrate (CM) and anhydrate (Cd), each viewed down the *b*-axis. One-dimensional hydrogen-bonded ribbons, common to both forms, exist in two different antiparallel orientations identified by green solid and dashed ovals. CM (102) is a low-rugosity plane. Structures for CM and Cd were constructed using refcodes CYTOSM11 and CYTSIN01, respectively.

CM crystallizes from aqueous solution as transparent plates with large {100} faces. A packing diagram of the structure appears in Figure 2, with water molecules shown in blue. Cytosine molecules assemble into polar one-dimensional ribbons along the *b*-axis through N···H–N and NH_2_··O hydrogen bonds. Solid and dashed green ovals identify the different ribbon orientations which are related by 180°. Adjacent ribbons π-stack into dense layers in the (100) plane, with planes related by translation along the *a*-axis. Water molecules connect adjacent cytosine ribbons and layers through NH_2_···O_w_ and O_w_–H··O hydrogen bonds.

When heated, CM dehydrates to a phase we refer to as Cd. Cd has the same structure as the anhydrate with the lowest calculated lattice energy. The dehydration process from CM to Cd involves a high degree of cooperativity where simultaneous water loss and ribbon rotation enables the dense layers of π-stacked *antiparallel* ribbons to become orthogonal dense π-stacked layers of *parallel* ribbons. Notably, the very flat (102) plane in CM must buckle to generate Cd. There is no similar low rugosity extended two-dimensional plane in Cd.

### Design Strategy

Our defect engineering strategy is based on the incorporation of low concentrations of planar dye molecules in CM hosts, with the underlying assumption that each included dopant effectively replaces multiple cytosine molecules in the same (102) plane. This is similar in concept to the “tailor-made additive” approach pioneered by Lahav and Leiserowitz.^27,28^ Dye substitution in this low-rugosity plane should pose the smallest disruption^29,30^ to the surrounding lattice, and therefore strongly preferred over other alternatives. A dozen different dyes (see Figure 1) with a variety of core structures and charges were initially examined in CM growth studies to identify the ones that include in the highest concentrations. Even the best dopants were expected to include in low-levels, though creating substitutional defects with dye molecules affords the downstream benefit of facilitating quantification through UV–vis spectroscopy. With an expected maximum inclusion of ∼1%, all CM-dye phases would have a chemical purity of ∼99% or higher. Though dopant molecules could potentially adopt multiple orientations within the CM (102) plane, at least some dyes would be forced to span multiple (100) layers given their dimensions. Since the dehydration process buckles the low-rugosity (102) plane, we reasoned that dye molecules residing within that plane would introduce an additional barrier to the dehydration process.

### Preparation of CM-Dye

Dye-doped crystals were prepared by slow evaporation of saturated aqueous cytosine solutions (4 mg/mL = 36.0 mM) with dye concentrations in the range of 0.001–2.5 mM (1–2500 μM). We refer to the resultant materials as CM-dye_*x*_ where *x* = the solution dye concentration in μM. Pure CM crystals deposit as thin, transparent rectangular plates that may grow to mm and cm sizes. With the addition of dye to the growth solution, the resulting crystals deposited in a variety of irregular morphologies depending on the dye and its solution concentration (Figures S1 and S2). In some cases, the morphology changes may in part be related to changes in the solution pH which has been shown to alter the relative growth rates of CM,^31,32^ though we did not attempt to control this variable.

Dye inclusion was obvious in many cases from simple visual inspection, though the color intensity of individual crystals within a given batch and between batches could vary. Dye molecules likely include as monomers, as solid-state UV–vis spectra of CM-dye crystals showed no evidence of higher order dye aggregates (Figure S3). None of the dye-doped crystals exhibit the kind of hourglass or Maltese cross patterning that has been reported in other systems.^33−35^ However, when rotated under polarized light, crystals exhibit color changes suggesting that that dye is oriented within the structure and not occlusions of growth solvent (Figure S4). PXRD confirmed that all CM-dye phases were phase pure and isomorphous with CM (Figure S5).

### Quantification of [Dye] in CM-Dye

The concentration of included dye in CM-dye as a function of concentration in the growth solution was determined by solution UV–vis. CM-dye crystals were rinsed in DI water to remove any residual surface dye, weighed, then re-dissolved in aqueous solution and the measured absorbance was compared against standard calibration curves. Solutions of dissolved CM-dye crystals all had λ_max_ values within <7 nm of prepared aqueous dye calibration standards. Absorbance from cytosine did not affect the measurements, since it has a λ_max_ = 267 nm,^36^ which is sufficiently far from the absorption max of the visible dyes tested.

**Figure 3:**
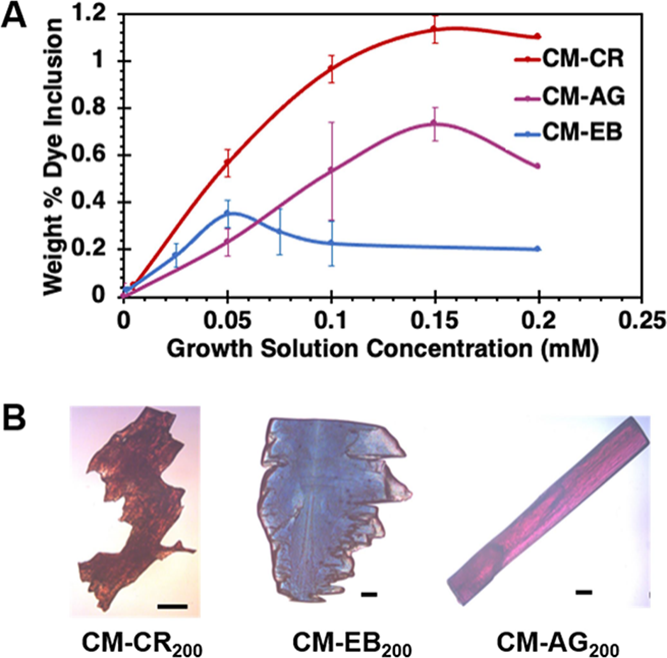
(A) Plot of included [dye] in CM-CR, CM-EB, and CM-AG as a function of growth solution [dye]. Measurements are based on UV–vis spectroscopy of dissolved crystals. (B) Representative crystals grown from 200 mM dye solutions. Maximum dopant loads included in CM-dye are: [CR] = 1.1 wt %, [AG] = 0.7 wt %, and [EB] = 0.3 wt %.

UV–vis measurements indicated the highest dopant loads in CM-dye were achieved with Congo Red (CR, max 1.1%), Azocarmine G (AG, max 0.7%), and Evan’s Blue (EB, max 0.3%) (Figure 3). CR, AG, and EB have other uses as histological stains^37−39^ and AG is also used in foodstuffs.^40^ Chrysoidine G (CG) could also reach dopant loads of 0.5%, however, this required 5–10× higher dye concentrations in the growth solution compared to the other potential dopants tested (Figure S6). For most other dyes examined, the dye concentration in CM-dye was at or below 0.1%.

In general, these results suggested that the inclusion of anionic dopants was preferred over neutral or cationic dyes in CM. We attribute this to the weak base nature of cytosine, as it can protonate to form cytosinium and hemicytosinium ions.^41^ The inclusion of these cations alongside the anionic dye molecules provides an easy means to maintain charge balance. The opposite selectivity was seen in previous studies on dye inclusion in uric acid, a weak acid, where the inclusion of cationic dyes was preferred over anionic ones.^35,42−44^

### CM-Dye Thermal Stability

Having established the isomorphous relationship between CM, CM-CR, CM-EB, and CM-AG and dopant concentration for each growth condition, the effect of each dopant on the thermal properties and process-induced dehydration were next investigated. A complementary set of methods was used, including hot stage microscopy, DSC, TGA, and PXRD.

Hot-stage microscopy experiments on CM^20,21^ showed that dehydration from single crystals typically starts from the outer edges, with opaque reaction fronts expanding inward over time until the entire crystal eventually darkens. The final product, Cd, is polycrystalline but retains the original plate morphology. However, observing the dehydration CM-CR, CM-EB, and CM-AG was more difficult owing to the strong color. Nevertheless, it appears that dehydration of these CM-dye crystals proceeds qualitatively the same as in pure CM, with dehydration initiating from the jagged rough edges of the crystals and propagating inward until the crystals fully darken (Figure S7).

Importantly, we previously showed that the DSC dehydration endotherms of as-grown (unground), hand-ground, and ball-milled CM had indistinguishable *T*_max_ values.^21^ This confirmed that by DSC, CM dehydration temperatures determined were independent of particle size. We reference *T*_max_ = 96 ± 4 °C as the value for hand-ground CM for comparisons to hand-ground CM-dye phases. For each type of doped material, CM-CR, CM-EB, and CM-AG, significant thermal stability gains were observed relative to CM. However, the magnitude of the increase in *T*_max_ was dependent on the specific dye and its concentration.

**Figure 4:**
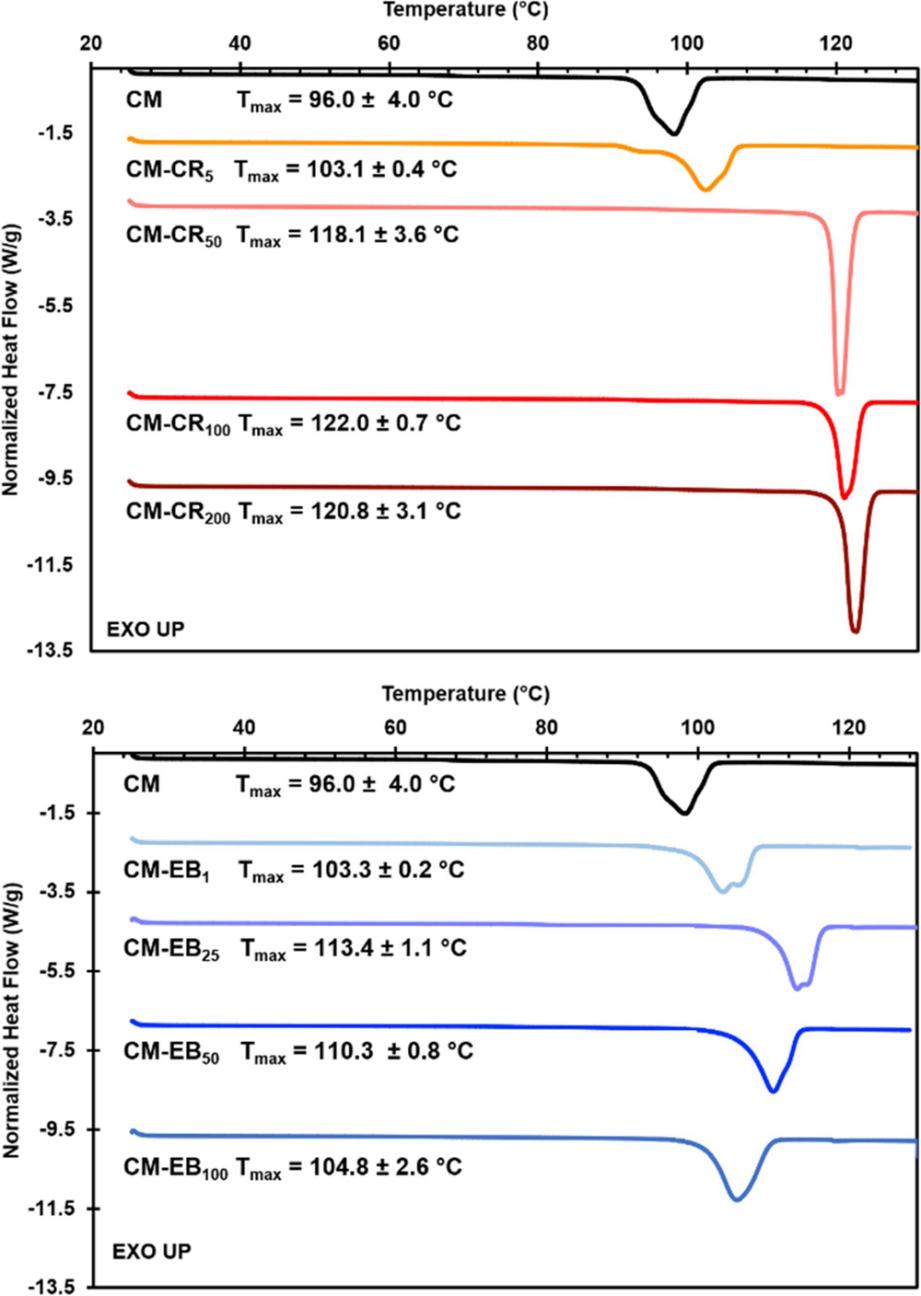
(top) DSC curves of CM-CR and (bottom) CM-EB grown from solutions with different dye concentrations. Hand-ground samples were heated in hermetically sealed pans at 5 °C/min. The *T*_max_ of the dehydration endotherm trends with the dopant load.

As Figure 3A shows, growth from solutions with [CR] = 5–150 mM yielded CM-CR with progressively higher dopant loads, though solutions with [CR] > 150 mM did not result in further increases. DSC thermograms for CM, CM-CR_5_, CM-CR_50_, CM-CR_100_, and CM-CR_200_ are compared in Figure 4. The *T*_max_ of the dehydration endotherm generally follows the same trend as the dopant concentration. Even at the lowest dopant loads (0.05% CR), CM-CR_5_ exhibited an increase ∼7 °C relative to pure CM. At the maximum dopant loads (1.1% CR), the *T*_max_ of CM-CR_100_ is ∼26 °C higher than that of pure CM. The *T*_max_ values for CM-CR_50_, CM-CR_100,_ and CM-CR_200_ are the same within experimental error, suggesting that dopant loads above 0.5% do not yield appreciable thermal stability gains but only decrease chemical purity. Nevertheless, for CM-CR_100_, which has a chemical purity of 99%, a 26 °C increase in dehydration temperature is quite remarkable.

For CM-EB, growth from solutions with increasing EB concentrations reached maximum dopant loading at 50 mM, and slightly lower inclusion levels from more concentrated solutions. Though somewhat counterintuitive, we suspect the slight decrease may be related to a reduction in the growth rate, which would favor the exclusion of impurities. A similar effect is seen for CM-AG, though the maximum dopant load in that case is reached at 150 mM. DSC thermograms for CM, CM-EB_1_, CM-EB_25_, CM-EB_50_, and CM-EB_100_ are also compared in Figure 4. The highest thermal stability gains were seen in CM-EB_25_ with a *T*_max_ = 113.4 ± 1.1 °C, which is ∼17.4 °C higher than CM. Again, the general trend in *T*_max_ seems to follow the concentration of included dopant. Thermal stability gains in CM-AG were more modest, with CM-AG_200_ (AG = 0.6%) reaching a maximum of *T*_max_ = 107.6 °C ± 0.5 °C (Figure S8). Although CM-CG could also be grown with up to 0.5% CG loads, the highest *T*_max_ observed was only 101.8 °C ± 0.7 °C (Figure S9).

TGA confirmed the water content in CM, CM-CR, CM-EB, and CM-AG were all equivalent within experimental error. The only exception was CM-CR_50_ which lost 13.4 ± 0.05 wt %, slightly under the calculated 13.9 wt % water content. There are no other thermal transitions in the TGA upon further heating to 250 °C. Cytosine typically begins to decompose at or above 300 °C. PXRD patterns of all dehydrated materials, Cd-dye, matched the expected pattern for Cd (refcode: CYTSIN01) (Figure S10).

### CM-Dye Dehydration Kinetics

**Figure 5:**
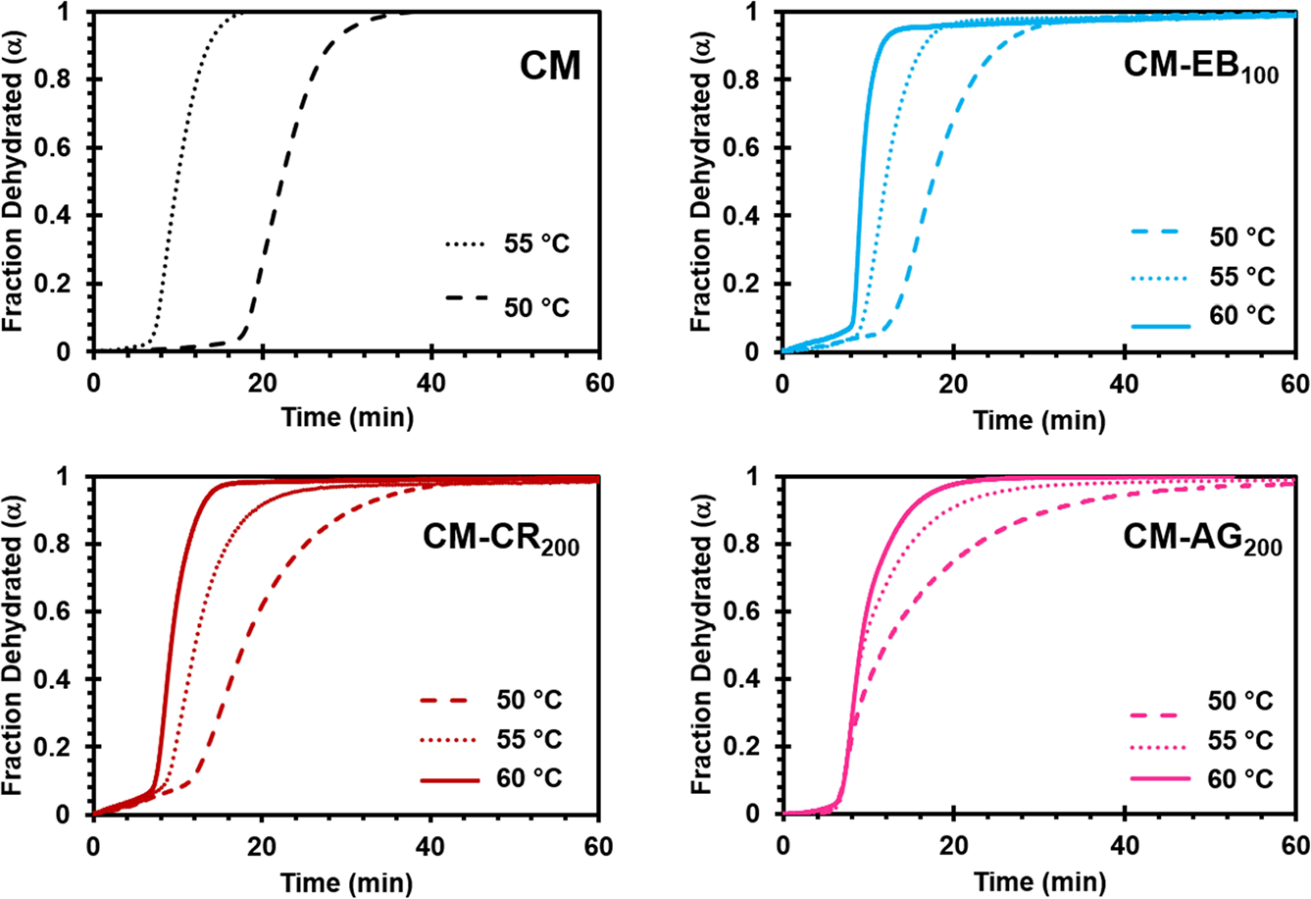
Representative TGA isothermal dehydration data for hand-ground samples of CM, CM-CR_200_, CM-EB_100_, and CM-AG_200_ at 50 °C (dashed), 55 °C (dotted), or 60 °C (solid).

To gain a deeper understanding of how the dopant inclusion impacts the CM dehydration kinetics, isothermal TGA experiments were performed in triplicate on three different hand-ground CM-dye samples at 50, 55, and 60 °C. In Figure 5, the reaction progress is plotted in terms of the fraction dehydrated (α) with respect to time for CM-CR_200_, CM-EB_100_, and CM-AG_200_ samples. As expected, dehydration is faster as the isothermal temperature is increased. Previous work indicated that the CM dehydration kinetics varied depending on how the sample was processed (unground, manually-ground, or milled).^21^ Samples shown in Figure 5 were prepared by the same experimentalist using a similar grinding rigor.

There are noticeable differences in the onset temperatures for dehydration in the CM-dye samples compared to pure CM. Both CM-EB_100_ and CM-CR_200_ appear to gradually lose 5–10% of their water content slowly before the rate suddenly accelerates. In the 50 °C data sets, the onset of dehydration in CM-AG_200_ appears earlier than in undoped CM, though it obviously takes far longer for the dehydration reaction to reach completion. Differences in the 55 °C data sets also generally show slower dehydration in the CM-dye samples. While CM has completely dehydrated after ∼16 min, the dehydration of CM-dye systems is only 80–85% complete in this time.

For a more quantitative comparison, data from the linear region of each CM-dye curve (0.1 < α < 0.7) were fit to several solid-state kinetic models^45,46^ (Tables S1 and S2) in an effort to identify the most probable rate-limiting step in the reaction. Seventeen models were evaluated based on the correlation coefficient (*R*^2^) to the data. Each CM-dye had an *R*^2^ > 0.99 for multiple models, but few with an *R*^2^ > 0.999. As there was no singular “best fit,” this model-fitting approach proved inconclusive. Since data from all samples had a reasonable fit to a generic first-order solid state reaction model, the rate constants (*k*) were determined using the equation *k* = −ln (1 – α)/*t* where *t* = time in minutes. CM dehydration at 50 and 55 °C proceeded with a *k* = 0.20 and 0.34 min^–1^, respectively. At the same temperatures, *k* decreased by 6–10% for CM-EB_100_, 25–44% for CM-CR_200_, and 51% for CM-AG_200_ (Table S3). These indicate a direct correlation between the dopant and the rate of conversion to the Cd lattice. With measurements at three temperatures, the activation energy (*E*_a_) could be determined from Arrhenius plots. Model-free Friedman methods,^47^ which enable the calculation of *E*_a_ at different time points, indicated a fairly consistent *E*_a_ over the linear region of the reaction coordinate. For the dye-doped materials, the average *E*_a_ = 108.6 ± 7.8 kJ/mol (CM-EB_100_), *E*_a_ = 131.3 ± 3.9 kJ/mol (CM-CR_200_), and *E*_a_ = 104.3 ± 10.6 kJ/mol (CM-AG_200_). In comparison, the same model-free methods yielded an *E*_a_ = 96.4 ± 1.7 kJ/mol for CM.^21^

### Time-Resolved Structural Changes

**Figure 6:**
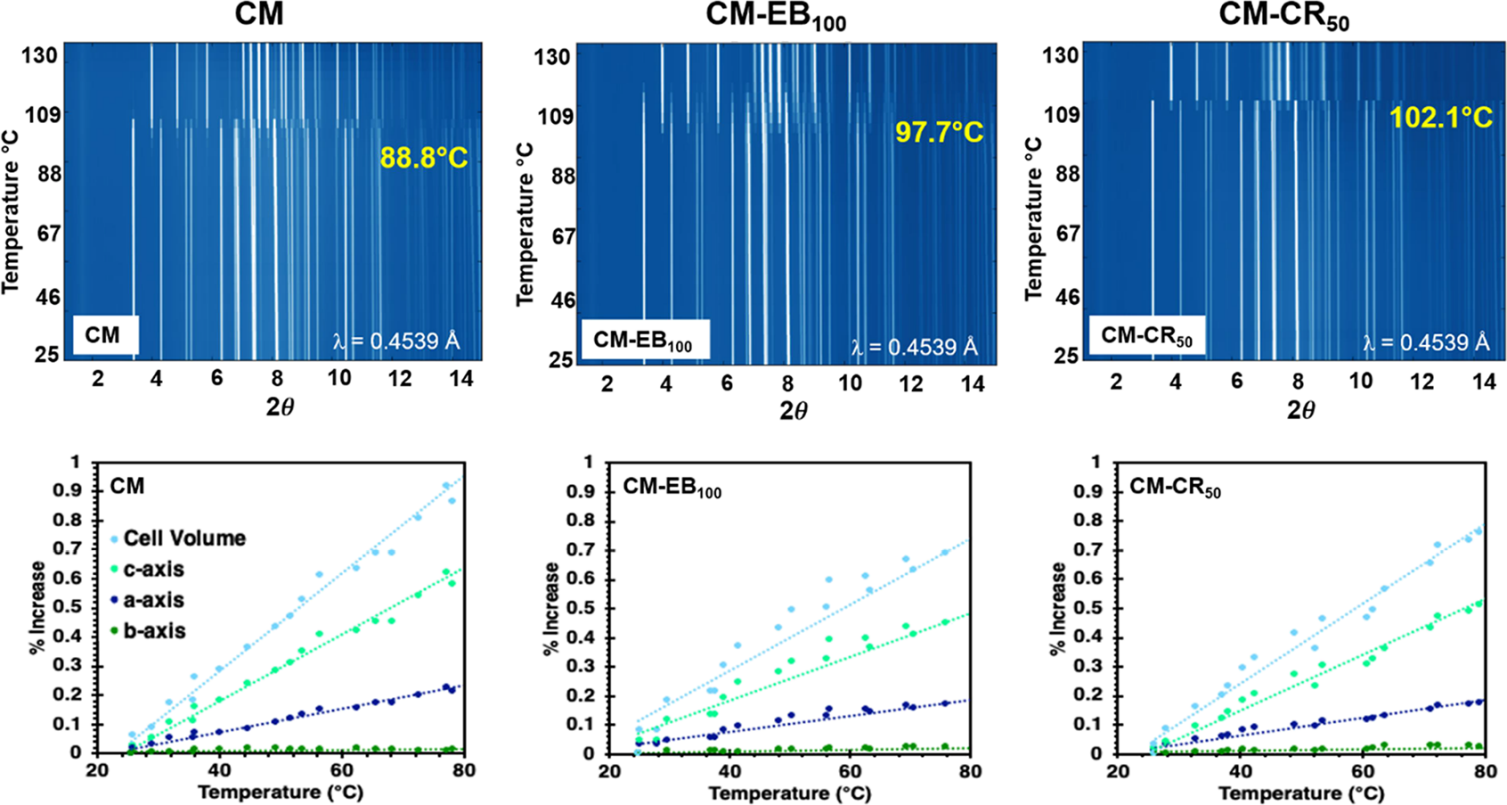
(top) Contour plots of multiple sPXRD patterns collected during the dehydration of CM, CM-EB_100_, and CM-CR_50_ when heated to 130 °C at 10 °C/min (RH = 0%). (bottom) Thermal expansion plots of CM, CM-EB_100_, and CM-CR_50_ based on the refinement of sPXRD patterns between 25 and 80 °C.

Structure changes that occur during the CM-dye to Cd-dye transformation were investigated using time-resolved synchrotron powder diffraction (sPXRD). In-situ sPXRD dehydration experiments were performed on phase-pure CM, CM-EB_100_, CM-CR_50_, and CM-CR_200_ by heating the samples at 10 °C/min under a controlled atmosphere (RH = 0%). Fast data acquisition methods enabled diffraction patterns to be collected every ∼20 s. Contour plots for CM, CM-EB_100_, CM-CR_50_, and CM-CR_200_, are shown in the top of Figures 6 and S12. In all experiments, dehydration led to only one crystalline product, with no evidence of other crystalline intermediates. In the dehydration of CM, peaks corresponding to Cd are first detectable at 88 °C and the reaction reaches completion quickly in about 2 min. In CM-EB_100_, CM-CR_50_, and CM-CR_200_, the product appearance is delayed to 97.7, 102.1, and 112.5 °C, respectively. The CM-EB_100_ sample also required a longer time to reach full-conversion. CM-CR samples do not show extended reaction times, though at higher temperatures one expects faster reaction kinetics.

In the dehydration of CM, all evidence indicated that water loss and Cd formation occur simultaneously via a cooperative mechanism, where ribbon rotation releases water and vice versa. Based on single crystal structures obtained between 100 and 295 K, the thermal expansion of CM was shown to be highly anisotropic, with the largest expansion occurring between the π-stacked 1-D ribbons (*c*-axis) within the dense (100) planes.^21^ Between 100 and 295 K, the cell volume and *c*-axis expand by ∼4% and ∼2.5%, respectively. We expected continued increases in these parameters up to the point where CM transforms to Cd. Sequential Pawley refinement of CM sPXRD patterns collected between 25 and 80 °C (298 and 353 K) confirmed the cell volume and *c*-axis expand by another ∼1.07 and ∼0.72% in this temperature range (Figure 6, lower left). This means that from 100 K to when dehydration occurs, the distance between the 1-D ribbons (*c*-axis) increases by just over 0.2 Å. We assume that ribbon rotation is at least in part facilitated by this increased separation, and also note that the π-stacks in Cd have a shorter repeat distance than those in CM.

The intentional creation of substitutional defects in the (102) planes was envisioned as a means to introduce a physical impediment to this ribbon rotation since the included dye molecules would be unable to buckle. There is other evidence that suggests the substitutional defects also alter the properties in more subtle ways. First, sequential refinement of sPXRD patterns from CM-EB_100_ and CM-CR_50_ collected between 25 and 80 °C (298 and 353 K) clearly show that doping alters the thermal expansion properties (Figure 6, lower middle and right plots). The changes in cell volume and *c*-axis in CM-EB_100_ and CM-CR_50_ are noticeably smaller than those in pure CM. With no evidence for other phase changes or water loss below 80 °C (Figure 4), shifts in parameter values must be due to thermal expansion. Assuming expansion along the *c*-axis is a prerequisite to ribbon rotation, materials with these designer defects would necessarily require higher temperatures or longer times for dehydration to occur.

**Figure 7:**
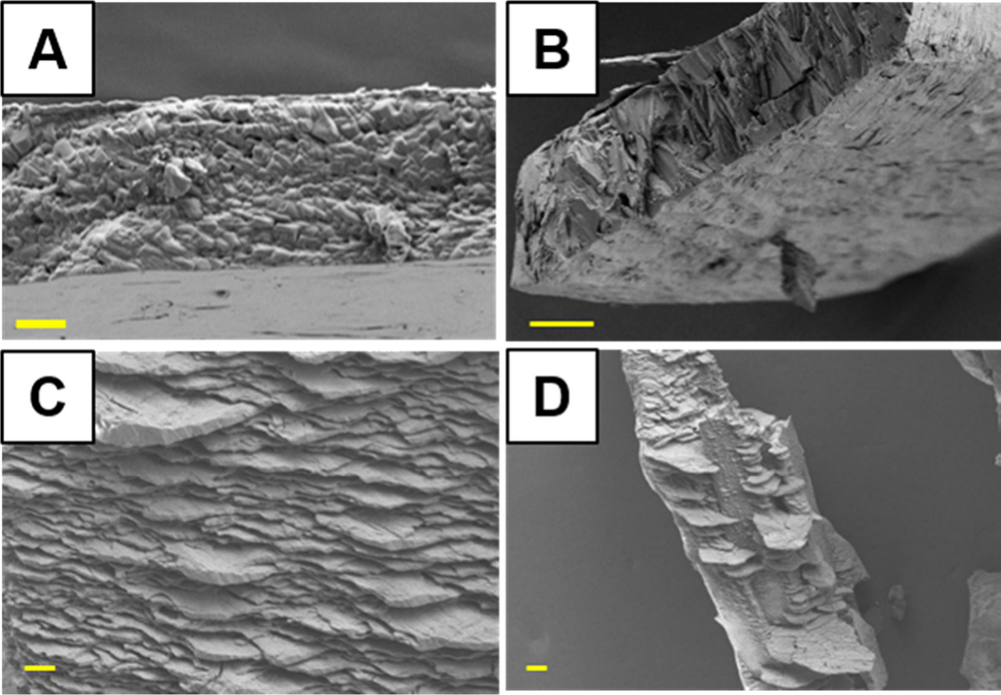
SEM images of products resulting from the dehydration of (A) CM, scale bar = 2 μm; (B) CM-EB_100_, scale bar = 10 μm; (C, D) CM-CR_200_, scale bars = 2 and 20 μm, respectively. Cd, Cd-EB_100_, and Cd-CR_200_ particles have noticeably different textures.

Second, dopant effects on the growth of the anhydrate are apparent based on morphological differences in SEM images of the dehydrated product (Figure 7). Individual CM crystals retain their macroscopic shape when dehydrated, though the product is polycrystalline. Cd and all Cd-dye have the same bulk structure (Figure S10), but their particle morphologies are noticeably different. Figure 7A is a typical SEM image of Cd, where the side faces have a granular texture and numerous cracks are observed on what was the plate face. CM-EB_100_ crystals also grow as plates, yet the side faces of the dehydrated Cd-EB_100_ have a very different texture with more angular features (Figure 7B). The topography of dehydrated CM-CR_200_ crystals is even more unusual, with thin plate-like Cd-CR_200_ particles seeming to emerge at an angle from the surface, almost reminiscent of shale (Figure 7C,D). Further work is needed to elucidate the details of how the dye molecules might alter the nucleation and growth of the anhydrate and potential property differences in these dehydrated phases.

## Conclusions

Hydrate–anhydrate phase transformations are complicated processes. Defining the environmental conditions where a hydrate is stable and then ensuring the material stays within an acceptable range is the typical means to minimize the risk of form conversion during manufacturing or storage. But environmental control need not be the only approach, and may not be the most feasible approach in all contexts. While other polymorphs, solvate, and cocrystal forms may be a way to improve properties, the structural changes are dramatic. Here, we have demonstrated “proof of concept” of an alternative strategy, one where subtle internal structure changes can be exploited to expand the hydrate stability range while still maintaining chemical purity of ∼99%. The success of this approach opens the door to a vast amount of unexplored phase space.

Admittedly, the known dehydration mechanism of CM was a considerable advantage in the conception of our defect-design strategy, yet even with this benefit, determining which dyes exhibit reasonable inclusion levels was, to some extent, a matter of trial-and-error. We expect this defect-engineering approach can be effective in other systems, though dopant selection and optimization will remain an issue until the rules which govern dopant inclusion and/or impurity rejection^48−50^ are better understood.

## Supporting Information Available

Optical micrographs; solid state UV–vis spectra for CM; representative CM-EB, CM-CR, CM- IC, CM-AG, and CM-ErB crystals; PXRD of hand-ground CM-dye; micrographs of CM-CG25, CM-CG50, CM-CG250, CM-CG1250, CM-CG2500; hot stage microscopy of CM-CR200, CM-EB100 and CM-AG200 crystals; DSC curves of CM, CM-AG50, and CM-AG200; DSC curves of CM, CM-CG25, CM-CG50, CM-CG250, CM-CG1250, and CM-CG2500; PXRD of the Cd-dye products resulting from dehydration of CM-dye; solid state reaction models and integral expressions used for kinetic analyses; model-free activation energies; comparison of contour plots for CM, CM-CR50, and CM-CR200; average regression parameters; temperature-dependent rate constant (PDF)
