# Supplementary material for: Enhancement of Hydrate Stability through Substitutional Defects
Source: Cryst Growth Des. 2023 Jun 28;23(8):5860–7. doi: 10.1021/acs.cgd.3c00457 (PMC10401670; doi:10.1021/acs.cgd.3c00457)
Supplement: Supplementary file 1 — cg3c00457_si_001.pdf [file cg3c00457_si_001.pdf]

Supplementary Information

Enhancement of Hydrate Stability through Substitutional Defects

Megan E. Fleming and Jennifer A. Swift*

Georgetown University, Department of Chemistry, 37th and O Sts NW, Washington, DC 20057-1227, United States

Table of Contents

	page
**Figure S1.** Optical micrographs of (A) CM, Scale bar = 1 cm, (B) CM-CR_{5}, CM-CR_{50}, CM-CR_{100}, CM-CR_{150}, CM-CR_{200}, (C) CM-AG_{5}, CM-AG_{50}, CM-AG_{100}, CM-AG_{150}, CM-AG_{200}, (D) CM-EB_{1}, CM-EB_{25}, CM-EB_{75}, CM-EB_{100}, CM-EB_{200}, Scale bar = 100 μm.	**3**
**Figure S2.** Optical micrographs of typical CM-dye crystals grown from aqueous dye solutions. Scale bars = 100 μm.	**3**
**Figure S3.** Representative solid state UV-Vis spectra for CM and each type of visibly colored CM-dye.	**4**
**Figure S4.** Representative CM-EB, CM-CR, CM- IC, CM-AG, and CM-ErB crystals grown from 0.05 mM dye solutions viewed under linearly polarized light. Crystals exhibit different absorption depending on the polarized light direction, indicating oriented inclusion. Arrow indicates direction of polarization. Scale bars = 100 μm.	**4**
**Figure S5.** PXRD of hand-ground CM-dye and the simulated PXRD from the single crystal structure (CYTOSM11) confirms all are isomorphous with CM and phase pure.	**5**
**Figure S6.** (top, left to right) Micrographs of CM-CG_{25}, CM-CG_{50}, CM-CG_{250}, CM-CG_{1250}, CM-$CG_{2500,}$ scale bar = 100 μm. (bottom) Included [CG] in CM-CG as a function of growth solution [dye]. Measurements are based on UV-Vis spectroscopy of dissolved crystals.	**5**
**Figure S7.** Hot stage microscopy of CM-CR_{200}, CM-EB_{100} and CM-AG_{200} crystals heated at 5 °C/min. Scale bars = 100 μm.	**6**

Figure S8. DSC curves of CM, CM-AG$_{50}$ and CM-AG$_{200}$. Hand ground samples were heated in hermetically sealed pans at 5 °C/min. **6**

Figure S9. DSC curves of CM, CM-CG$_{25}$, CM-CG$_{50}$, CM-CG$_{250}$, CM-CG$_{1250}$ and CM-CG$_{2500}$. Hand ground samples were heated in hermetically sealed pans at 5 °C/min. **7**

Figure S10. PXRD of the Cd-dye products resulting from dehydration of CM-dye. Data are compared against CM dehydration product, Cd, and the simulated powder pattern from the single crystal structure of anhydrous cytosine (CYTSIN01). **7**

Table S1. Solid state reaction models and integral expressions used for kinetic analyses **8**

Table S2. Average regression parameters from TGA isothermal dehydration of ground CM-CR$_{200}$, CM-EB$_{100}$ and CM-AG$_{200}$. Correlation coefficients (R^2) are listed for the data fit to each solid-state reaction models. **9**

Table S3. Temperature-dependent rate constant (min^{-1}) for CM dehydration, assuming first-order (F1) solid state reaction kinetics. **10**

Figure S11. Model-free activation energies as a function of reaction progress for CM-EB$_{100}$, CM-CR$_{200}$ and CM-AG$_{200}$. **10**

Figure S12. Comparison of contour plots for CM, CM-CR$_{50}$ and CM-CR$_{200.}$ Each sample was heated at 10 °C/min at RH = 0%. **11**

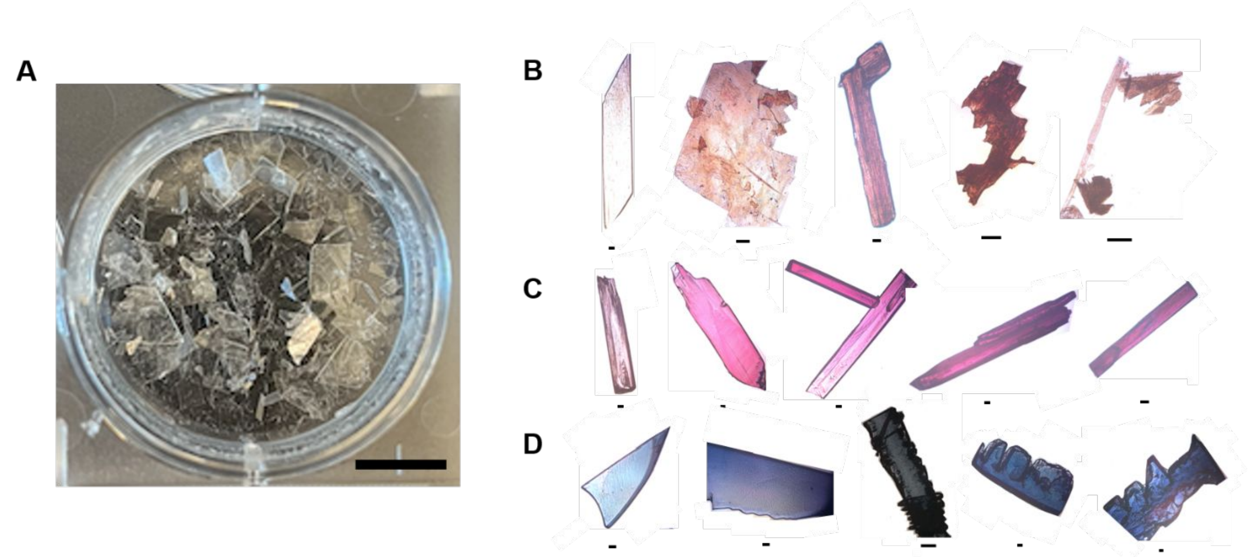

Figure S1. Optical micrographs of (A) CM, Scale bar = 1 cm, (B) CM-CR$_5$, CM-CR$_{50}$, CM-CR$_{100}$, CM-CR$_{150}$, CM-CR$_{200}$, (C) CM-AG$_5$, CM-AG$_{50}$, CM-AG$_{100}$, CM-AG$_{150}$, CM-AG$_{200}$, (D) CM-EB$_1$, CM-EB$_{25}$, CM-EB$_{75}$, CM-EB$_{100}$, CM-EB$_{200}$, Scale bar = 100 μm.

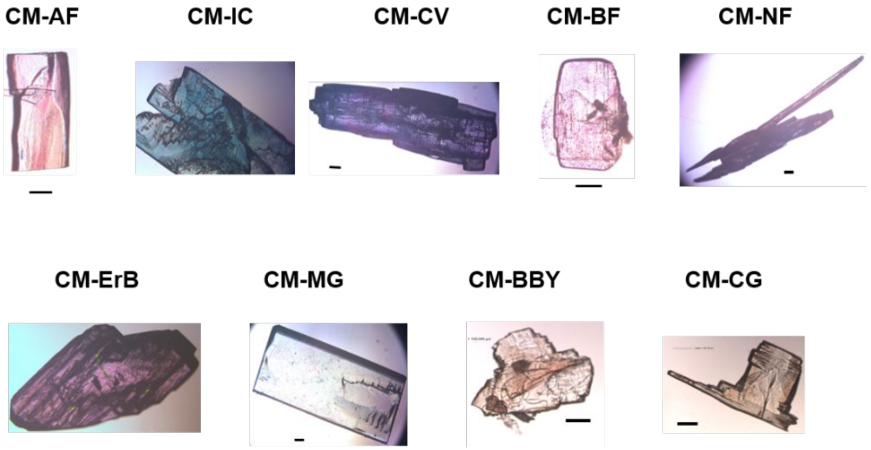

Figure S2. Optical micrographs of typical CM-dye crystals grown from aqueous dye solutions. Scale bars = 100 μm.

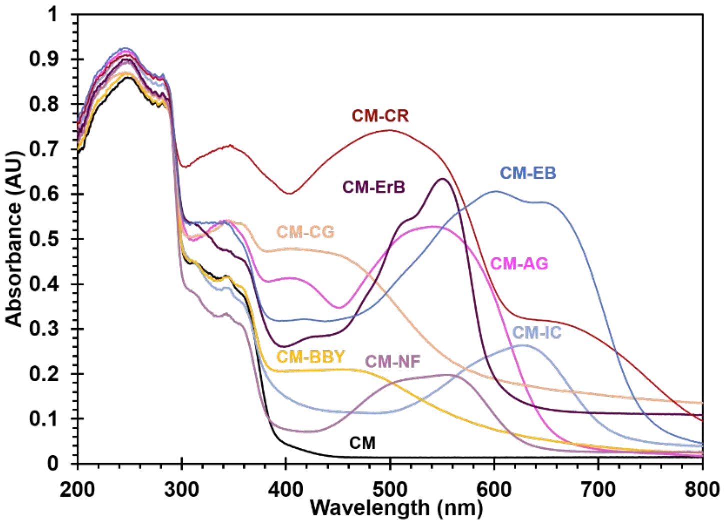

Figure S3. Representative solid state UV-Vis spectra for CM and each type of visibly colored CM-dye.

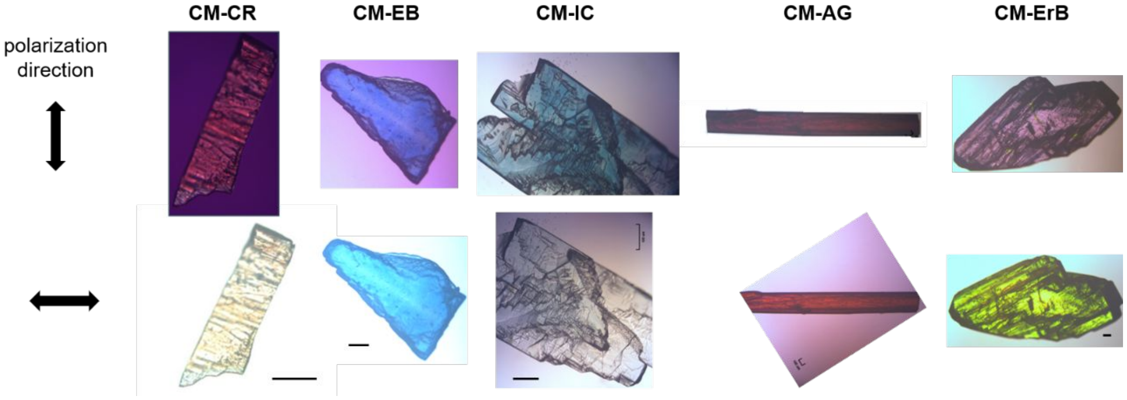

Figure S4. Representative CM-EB, CM-CR, CM- IC, CM-AG, and CM-ErB crystals grown from 0.05 mM dye solutions viewed under linearly polarized light. Crystals exhibit different absorption depending on the polarized light direction, indicating oriented inclusion. Arrow indicates direction of polarization. Scale bars = 100 µm.

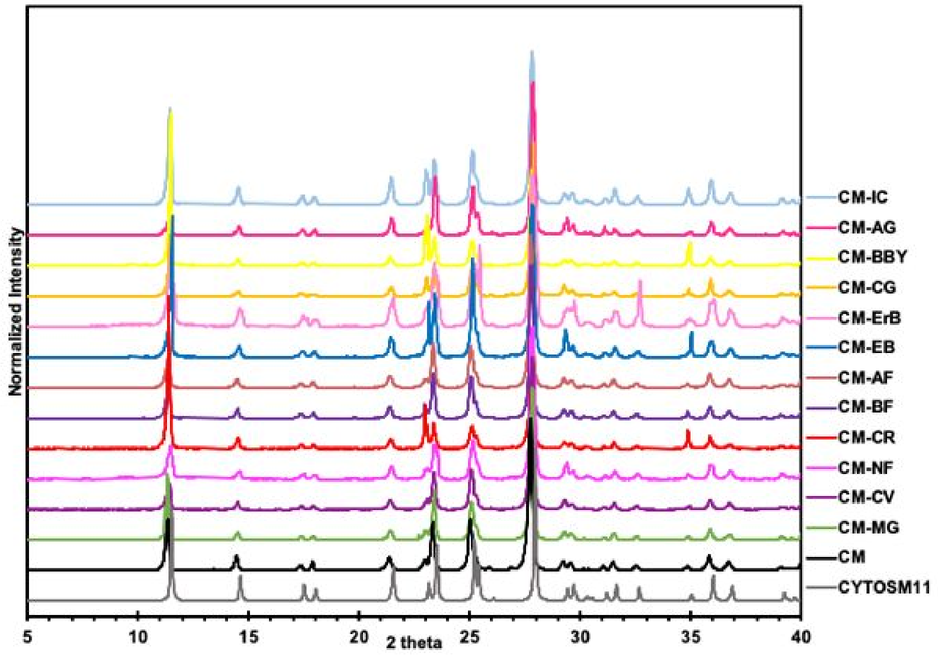

Figure S5. PXRD of hand-ground CM-dye and the simulated PXRD from the single crystal structure (CYTOSM11) confirms all are isomorphous with CM and phase pure.

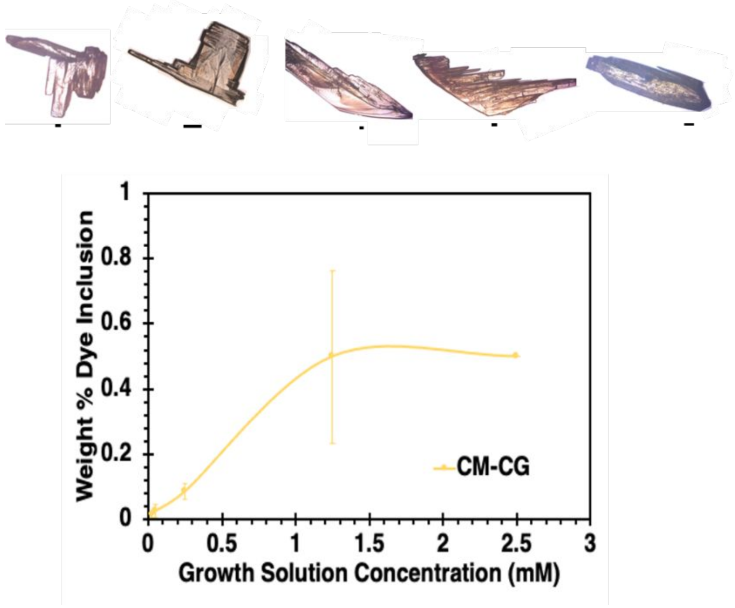

Figure S6. (top, left to right) Micrographs of $CM\text{-}CG_{25}$, $CM\text{-}CG_{50}$, $CM\text{-}CG_{250}$, $CM\text{-}CG_{1250}$, $CM\text{-}CG_{2500,}$ scale bar = 100 µm. (bottom) Included [CG] in CM-CG as a function of growth solution [dye]. Measurements are based on UV-Vis spectroscopy of dissolved crystals.

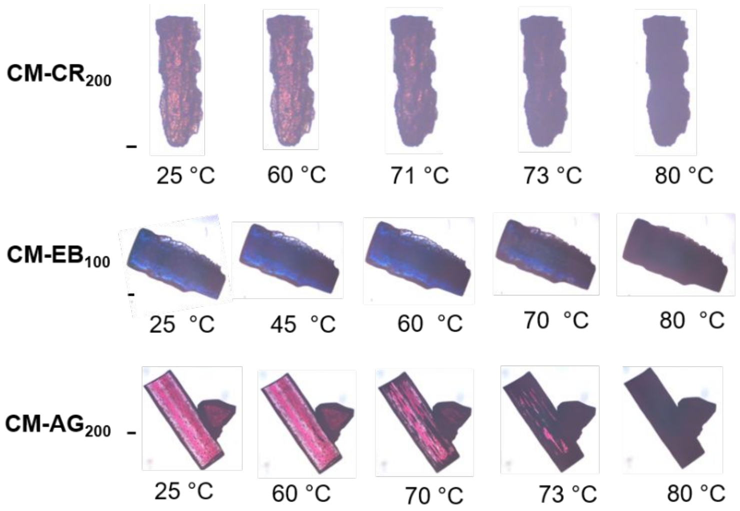

Figure S7. Hot stage microscopy of CM-CR_{200}, CM-EB_{100} and CM-AG_{200} crystals heated at 5 °C/min. Scale bars = 100 µm.

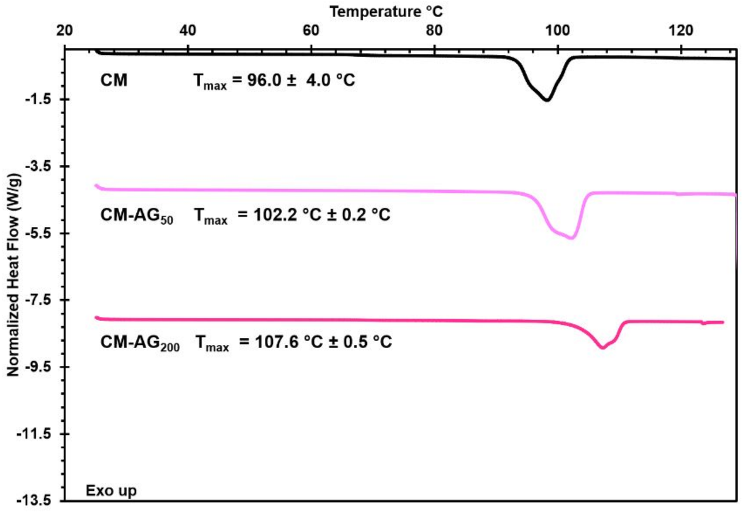

Figure S8. DSC curves of CM, CM-AG_{50} and CM-AG_{200}. Hand ground samples were heated in hermetically sealed pans at 5 °C/min.

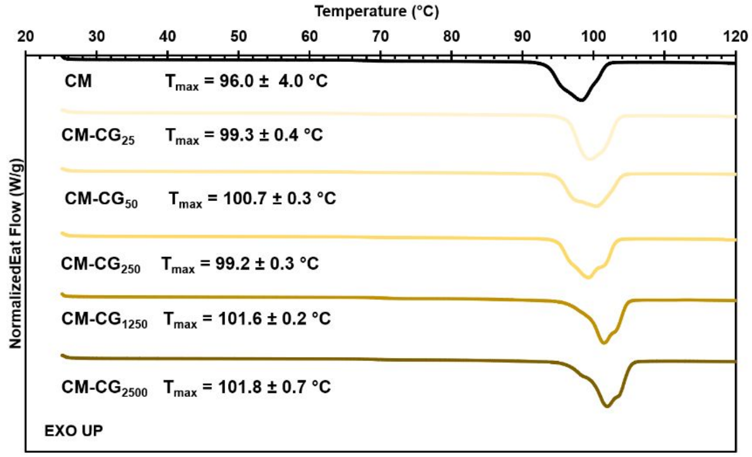

Figure S9. DSC curves of CM, CM-CG_{25}, CM-CG_{50}, CM-CG_{250}, CM-CG_{1250} and CM-CG_{2500}. Hand ground samples were heated in hermetically sealed pans at 5 °C/min.

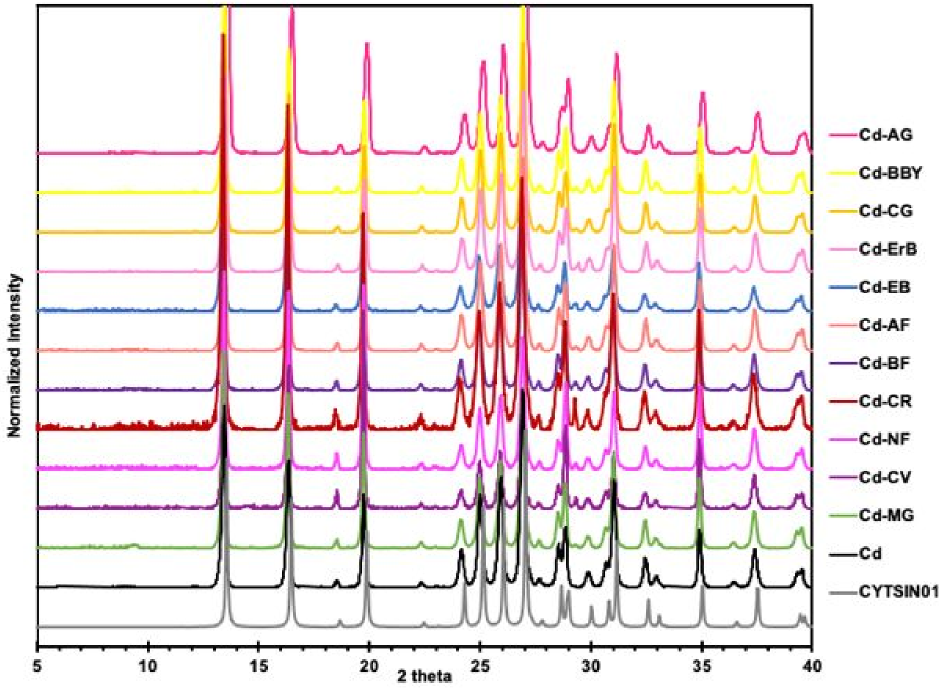

Figure S10. PXRD of the Cd-dye products resulting from dehydration of CM-dye. Data are compared against CM dehydration product, Cd, and the simulated powder pattern from the single crystal structure of anhydrous cytosine (CYTSIN01).

Table S1. Solid state reaction models and integral expressions used for kinetic analyses.[a-c]

**Dehydration Models**	**Integral Equation** $g(\alpha) = kt$
Nucleation & Growth	
1D growth of nuclei (Avrami-Erofeyev)(**A2**)	$(-\ln(1-\alpha))^{0.5}$
2D growth of nuclei (Avrami-Erofeyev) (**A3**)	$(-\ln(1-\alpha))^{1/3}$
3D growth of nuclei (Avrami-Erofeyev) (**A4**)	$(-\ln(1-\alpha))^{1/4}$
Random nucleation (Prout-Tompkins) (**B1**)	$\ln(\alpha/(1-\alpha)) + e^{\alpha}$
Power law (n = 1/2) (**P2**)	$\alpha^{1/2}$
Power law (n = 1/3) (**P3**)	$\alpha^{1/3}$
Power law (n = 1/4) (**P4**)	$\alpha^{1/4}$
Geometrical Contraction	
Zero-order (**R1**)	α
2D phase boundary (**R2**)	$1-(1-\alpha)^{1/2}$
3D phase boundary (**R3**)	$1-(1-\alpha)^{1/3}$
Diffusion	
1D diffusion (**D1**)	α^{2}
2D diffusion (**D2**)	$(1-\alpha)*(\ln(1-\alpha))+\alpha$
3D diffusion (Jander) (**D3**)	$(1-(1-\alpha)^{1/3})^{2}$
3D diffusion (Ginstling-Brounshtein) (**D4**)	$(1-(2/3)*\alpha)-(1-\alpha)^{2/3}$
Reaction Order	
First-order (**F1**)	$-\ln(1-\alpha)$
Second-order (**F2**)	$(1/(1-\alpha))-1$
Third-order (**F3**)	$(1/2)*(((1-\alpha)^{-2})-1)$

[a]Khawam, A.; Flanagan, D. R., *J. Pharm. Sci.* **2006,** *95* (3), 472-498; [b]Khawam, A.; Flanagan, D. R., *J. Phys. Chem. B* **2006,** *110* (35), 17315-17328; [c]Galwey, A. K., *Thermochim. Acta* **2000,** *355*, 181-238.

CM-Dye			Nucleation & Growth Models							Geometrical Contraction Models			Diffusion Models				Reaction Order Models		
			A2	A3	A4	P1	P2	P3	P4	R1	R2	R3	D1	D2	D3	D4	F1	F2	F3
$CM\text{-}CR_{200}$	50 C	R^2	0.9917	0.9792	0.9704	0.9820	0.9403	0.9212	0.9104	0.9785	0.9961	0.9978	0.9896	0.9789	0.9520	0.9714	0.9944	0.9285	0.8094
	65 C	R^2	0.9910	0.9801	0.9722	0.9833	0.9376	0.9193	0.9090	0.9747	0.9933	0.9948	0.9870	0.9762	0.9465	0.9681	0.9895	0.9100	0.7743
	60 C	R^2	0.9912	0.9822	0.9763	0.9858	0.9371	0.9243	0.9174	0.9673	0.9927	0.9971	0.9947	0.9961	0.9817	0.9932	0.9988	0.9483	0.8416
$CM\text{-}EB_{100}$	50 C	R^2	0.9949	0.9846	0.9768	0.9874	0.9450	0.9268	0.9164	0.9807	0.9968	0.9975	0.9887	0.9759	0.9446	0.9671	0.9909	0.9087	0.7719
	55 C	R^2	0.9921	0.9797	0.9709	0.9829	0.9350	0.9150	0.9037	0.9758	0.9959	0.9978	0.9915	0.9813	0.9522	0.9733	0.9932	0.9154	0.7811
	60 C	R^2	0.9831	0.9666	0.9560	0.9711	0.9118	0.8902	0.8784	0.9593	0.9897	0.9953	0.9926	0.9917	0.9736	0.9876	0.9985	0.9437	0.8263
$CM\text{-}AG_{200}$	50 C	R^2	0.9565	0.9330	0.9190	0.9377	0.8849	0.8586	0.8442	0.9449	0.9767	0.9843	0.9959	0.9997	0.9937	0.9988	0.9950	0.9893	0.9378
	55 C	R^2	0.9555	0.9348	0.9227	0.9406	0.8871	0.8651	0.8532	0.9400	0.9725	0.9805	0.9923	0.9987	0.9955	0.9989	0.9922	0.9905	0.9425
	60 C	R^2	0.9862	0.9798	0.9761	0.9825	0.9596	0.9527	0.9490	0.9770	0.9902	0.9933	0.9961	0.9991	0.9975	0.9992	0.9978	0.9958	0.9725
CM	50 C (1)	R^2	0.9989	0.9931	0.9882	0.9933	0.9809	0.9685	0.9610	0.9991	0.9983	0.9958	0.9733	0.9536	0.9250	0.9446	0.9877	0.9379	0.8588
	50 C (2)	R^2	0.9991	0.9946	0.9905	0.9952	0.9831	0.9726	0.9662	0.9976	0.9965	0.9942	0.9701	0.9516	0.9248	0.9431	0.9864	0.9393	0.8639
	50 C (3)	R^2	0.9937	0.9846	0.9782	0.9857	0.9670	0.9532	0.9452	0.9918	0.9980	0.9981	0.9853	0.9734	0.9539	0.9675	0.9955	0.9641	0.9034
	55 C (1)	R^2	0.9963	0.9878	0.9816	0.9884	0.9719	0.9577	0.9494	0.9963	0.9998	0.9989	0.9829	0.9667	0.9416	0.9589	0.9936	0.9520	0.8789

Table S2. Average regression parameters from TGA isothermal dehydration of ground $CM\text{-}CR_{200}$, $CM\text{-}EB_{100}$ and $CM\text{-}AG_{200}$. Correlation coefficients (R^2) are listed for the data fit to each solid-state reaction models. Those with R^2 values ≥ 0.99 are shaded, and those with $R^2 > 0.999$ are a darker shade. The data for CM in the blue box are taken from Fleming, M. E.; Watts, T. A.; McKenna, K. A.; Miehls, E. K.; Swift, J. A., *Cryst. Growth Des.* **2022**, *17*, 19.

Table S3. Temperature-dependent rate constant (min^{-1}) for CM dehydration, assuming first-order (F1) solid state reaction kinetics.

	CM	CM-EB$_{100}$	CM-CR$_{200}$	CM-AG$_{200}$
**50 °C**	0.20	0.182(8)	0.112(1)	0.099(3)
**55 °C**	0.34	0.319(9)	0.255(8)	0.167(7)
**60 °C**	N/A	0.638(6)	0.521(4)	0.323(5)

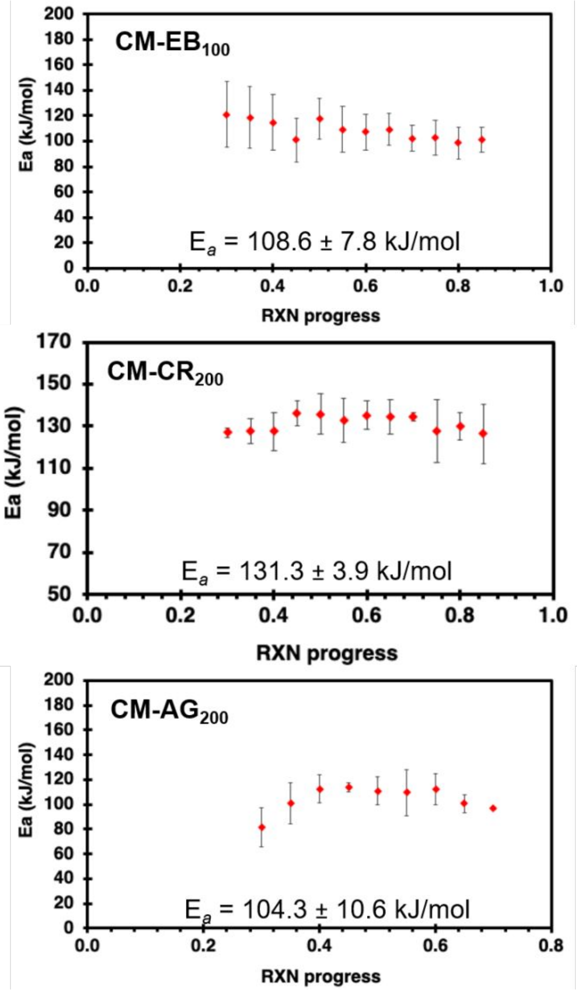

Figure S11. Model-free activation energies as a function of reaction progress for CM-EB$_{100}$, CM-CR$_{200}$ and CM-AG$_{200}$.

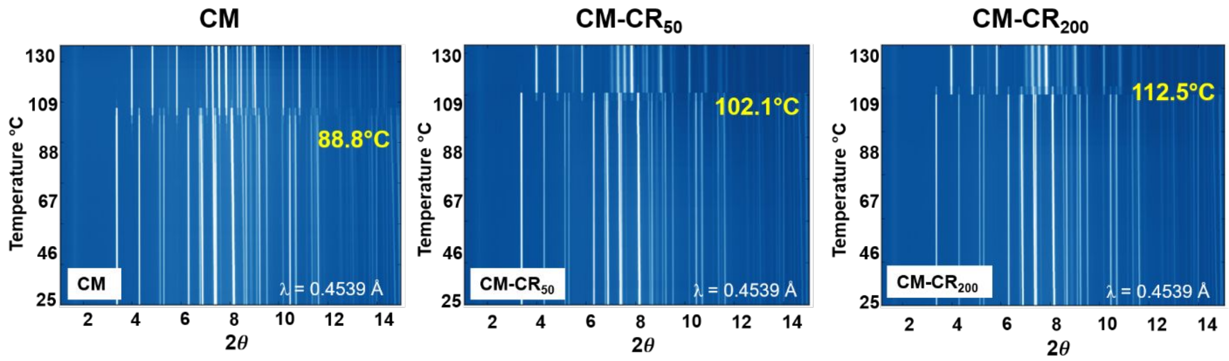

Figure S12. Comparison of contour plots for CM, CM-CR_{50} and CM-$CR_{200.}$ Each sample was heated at 10 °C/min at RH = 0%.
